# Biliary Dyskinesia in Children and Adolescents: A Mini Review

**DOI:** 10.3389/fped.2020.00122

**Published:** 2020-03-24

**Authors:** David A. Simon, Craig A. Friesen, Jennifer V. Schurman, Jennifer M. Colombo

**Affiliations:** ^1^Department of Pediatrics, Children's Mercy Kansas City, Kansas City, MO, United States; ^2^Division of Pediatric Gastroenterology, Children's Mercy Kansas City, Kansas City, MO, United States; ^3^Division of Developmental and Behavioral Sciences, Children's Mercy Kansas City, Kansas City, MO, United States

**Keywords:** biliary dyskinesia, cholescintigraphy, cholecystectomy, cholecystitis, gallbladder dysfunction

## Abstract

**Introduction:** While functional gallbladder disorder is a well-recognized and defined condition in adults, its pediatric analog, biliary dyskinesia, lacks uniformity in diagnosis. Yet, biliary dyskinesia is among the most common conditions resulting in cholecystectomy in youth and its frequency continues to rise. The primary aims of the current review were assess the efficacy of cholecystectomy in children diagnosed with biliary dyskinesia and the utility of cholescintigraphy in predicting outcomes.

**Results:** All previous studies assessing outcomes in youth with biliary dyskinesia have been retrospective and therefore of low quality. There is a lack of uniformity in patient selection. Short term follow-up reveals partial response in 63.4–100% with complete resolution in 44.2–100%. Only 4 studies have reported long-term outcomes with complete symptom resolution in 44–60.7%. The published research generally indicates that the gallbladder ejection fraction (GBEF) as determined by cholescintigraphy lacks utility in predicting cholecystectomy outcome utilizing the commonly used cut-off values. There are data suggesting that more extreme cut-off values may improve the predictive value of GBEF.

**Conclusion:** There is a lack of consensus on the symptom profile defining biliary dyskinesia in youth and current literature does not support the use of cholescintigraphy to select patients for cholecystectomy. There is a substantial portion of pediatric patients diagnosed with biliary dyskinesia who do not experience long-term benefit from cholecystectomy. Well-designed prospective studies of surgical outcomes are lacking. Increasing the uniformity in patient selection, including both symptom profiles and cholescintigraphy results, will be key in understanding the utility of cholecystectomy for this condition.

## Introduction

Functional gallbladder disorder is a well-recognized functional gastrointestinal disorder (FGID) in adults with well-accepted diagnostic criteria as defined by the Rome IV working group (1). The criteria characterizes “biliary pain” and defines functional gallbladder disorder as biliary pain in the absence of gall stones or structural pathology. Biliary pain is very specifically defined as epigastric or right upper quadrant (RUQ) pain which builds up to a steady level and lasts 30 min or longer, occurs at different intervals (not daily), is severe enough to interrupt daily activities, and is not related to bowel movements nor significantly relieved by postural change or acid suppression (1). Criteria listed as supportive of the diagnosis include a decreased gallbladder ejection fraction (GBEF) on cholescintigraphy and normal laboratory tests including liver function tests, amylase, and lipase. While Rome IV does not recognize this disorder in children and adolescents, biliary dyskinesia is a term that has traditionally been used to define an analogous, although less well-defined, condition in pediatrics.

Once biliary dyskinesia is diagnosed, the usual treatment is cholecystectomy as there is no accepted medical treatment specific to altering gallbladder function (2, 3). The rates of cholecystectomy for pediatric biliary dyskinesia continue to rise in the United States as numerous papers report positive outcomes with little to no surgical complications (4–10). Biliary dyskinesia is now among the most common conditions resulting in cholecystectomy in children and adolescents (11–13). However, long-term follow-up data suggests that certain patients undergoing surgical intervention for biliary dyskinesia will re-develop symptoms (14). It remains difficult for physicians to predict which patients will achieve long-term benefits from surgery.

This article represents a review of the most current information on biliary dyskinesia in children, particularly outcomes following cholecystectomy and the utility of cholescintigraphy in predicting short- and long-term clinical outcomes. Additionally, we assessed pathologic findings to determine the range of findings and whether these findings are similar to those reported in other pediatric FGIDs associated with pain.

## Symptoms

Although adult Rome IV criteria specifically define the location and characteristics of biliary pain, this definition has not been adopted in the pediatric criteria. While abdominal pain is the predominant symptom in nearly all studies of pediatric biliary dyskinesia, there is variability in the description of the pain or more frequently, little description of the pain. In a systematic review involving 1,833 youth with biliary dyskinesia, Sanntucci et al. found that RUQ pain was reported in 40% and epigastric pain in 17% leaving a significant proportion not fulfilling adult Rome criteria for biliary pain (15). Also at odds with Rome criteria, 61% reported daily pain (15). An additional 1,178 pediatric patients have been reported in the literature since the previous systematic review and report similar variability (14, 16–18). In the largest of these studies, a recent multicenter study involving 16 institutions and 678 patients, Cairo et al. reported RUQ pain in 76.7% and postprandial pain in 71.4% of pediatric patients diagnosed with biliary dyskinesia (16). For most previous studies, biliary dyskinesia was diagnosed by the presence of abdominal pain with slow gallbladder emptying demonstrated by cholescintigraphy (discussed below). This is problematic as slow gallbladder emptying can also be seen in association with a variety of other more common conditions associated with abdominal pain including functional dyspepsia, irritable bowel syndrome, and constipation (19–21). Given the variability in clinical response to cholecystectomy as discussed below, the existing literature points to the need to better define the characteristics of biliary pain in youth and assess whether a specific clinical profile increases the likelihood of benefit from surgery while minimizing risks and costs.

## Cholescintigraphy

The adult criteria for functional gallbladder disorder have evolved with cholescintigraphy transitioning from “diagnostic” to “supportive of the diagnosis,” based on accumulation of sufficient data to support this change. Two systematic reviews and one systematic review with meta-analysis generally do not support the use of cholescintigrahpy to determine gallbladder ejection fraction (GBEF) as a method to select adult patients for cholecystectomy (22–24). There is no difference in symptomatic response to cholecystectomy comparing those with low GBEF to those with normal GBEF (22, 24, 25).

In children and adolescents, cholescintigraphy to determine GBEF is generally performed to diagnose biliary dyskinesia, but it is not clear to what degree it drives patient selection for, or predicts patient response to, cholecystectomy. Seven studies assessing GBEF at cut-offs most commonly utilized to define delayed gallbladder emptying found no association between an abnormal GBEF and improved clinical response to cholecystectomy (7, 8, 14, 18, 25–27). In contrast, Krishna et al. reported that both GBEF <35% and pain reproduction during the test were associated with higher rates of symptom resolution (17). Mahida et al. reported a positive predictive value of ~80% for GBEF in identifying patients with a good clinical outcome, although the clinical cutoff was not provided (28). There are conflicting results on whether clinical response is better predicted by even lower GBEFs. Four studies have evaluated other GBEF cut-off values in predicting clinical outcomes (5, 29–31). In 2 studies, a GBEF <15% predicted better clinical outcome while 2 others found no relationship between GBEF <15% and better outcomes (5, 29–31). Lyons et al. reported that GBEF <11% was associated with better outcomes (30). Some studies have suggested that cholescintigraphy may have value beyond measuring GBEF, at least in adults. In adults, symptom reproduction with cholecystokinin (CCK) infusion is a better predictor of a beneficial response to cholecystectomy than GBEF (25). A single pediatric study found that cholecystectomy outcomes were not associated with symptom re-production (32). It should also be noted that pain with CCK does not appear to be specific to gallbladder dysfunction as pain is reproduced with CCK infusion in over half of adult patients with functional dyspepsia who report upper abdominal pain routinely associated with bloating and satiety (33). Overall, the current literature remains inconsistent and, thus, does not support the use of cholescintigraphy to select patients with suspected biliary dyskinesia for cholecystectomy.

There are many factors that could account for the lack of clear association between findings from cholescintgraphy and surgical outcomes. A more technical issue is that there is wide variety in the protocols employed for cholescintigraphy utilized to assess gallbladder emptying, as well as variability in defining normal cut-off values, as noted in the review of literature above. These issues likely lead to varying results from center to center (16). There also are patient-specific issues which may affect gallbladder emptying, including associated diseases and concomitant medications which are not always considered in interpreting results of cholescintigraphy. For example, low GBEF can be seen in diabetes, celiac disease, and Helicobacter pylori-associated gastritis (12). The slow gallbladder emptying associated with celiac disease reverts to normal on a gluten-free diet (34). Proton pump inhibitors, which are commonly prescribed empirically in patients with upper abdominal pain are associated with decreased GBEF in over 50% of patients while prokinetic medications (cisapride and erythromycin) have been shown to increase gallbladder emptying (35–37). In addition, although pediatric data are not available, an abnormal GBEF is seen in 15–25% of healthy adults (12). Conversely, initially abnormal GBEF in symptomatic patients has been shown to be normal on re-test in 23% of adult patients (38). Another adult study of 30 patients with an abnormal GBEF found that 16 of the 30 were normal on repeat (39). Similar findings have been reported in a single pediatric study where 12 patients had repeat cholecystingraphy, 58% had one normal and one abnormal study (9). Finally, the results of the test are likely not independent of the decision to pursue surgery, thus confounding any results. Individuals with a single abnormal GBEF or those with an abnormal GBEF with a potential disease- or medication-related cause may not require surgical intervention; it is unclear how these patient groups are being included or excluded in retrospective series of surgery. To date, no randomized clinical trials of cholecystectomy have been reported.

Given all of the above issues, as well as the lack of uniformity in the clinical symptoms defining biliary dyskinesia in children and adolescents and that symptoms likely affect the decision to perform cholecystingraphy in the first place, the value of cholescintigraphy in clinical decision making is not well-characterized. There is a need to understand and standardize the clinical phenotype, which may indicate symptoms on the basis of gallbladder dysfunction in youth, and then to assess the ability of cholescintigraphy to predict clinical outcomes in this more targeted population to determine if additional (or other) selection criteria are warranted.

## Cholecystectomy Outcome

In the last 15 years, there have been 19 pediatric studies with sample sizes of at least 30 reporting cholecystectomy outcomes in over 2,500 patients under 21 years of age with biliary dyskinesia. These are summarized in Table 1. Although variable from study to study, there is sometimes a high rate of reported symptom improvement in youth following cholecystectomy for biliary dyskinesia. Based on single center studies, this is at least true in the short term where a partial clinical response is reported in 63.4–100% (median 83.5%) and complete symptom resolution in 44.2–100% (median 70.5%). In the large multicenter study of Cairo et al. persistent symptoms were seen in 48.5% of patients but with significant variability between centers (16). The short-term response needs to be interpreted with caution as all of the studies were uncontrolled, retrospective in nature, and therefore do not account for spontaneous remission or a possible placebo-effect, as well as a variety of other issues discussed in further detail below. The long-term benefit is even less clear. Only four studies have evaluated long term outcomes. A partial clinical response is reported in 74–85% (2 studies) and complete symptom resolution in 44–60.7% (3 studies) (6, 25, 28, 30). In all but one of these studies, only 26–53% of patients were available for long term follow-up assessment (6, 25, 28, 30). The other study had 81% of patients available for assessment but defined long term as only >4 weeks post-operative (17).

**Table 1:** Patient outcomes following cholecystectomy for biliary dyskinesia.

**First author**	**No. of patients undergoing cholecystectomy**	**Patients available for short-term follow-up**	**Time until short-term follow-up**	**No. of patients with short-term complete clinical response (%)**	**Patients available for long-term follow-up**	**Duration of long-term follow-up (mean)**	**No. of patients with long-term complete clinical response (%)**
Carney et al. (31)	51				38	4.3 years	27 (71)
Vegunta et al. (6)	62	62	2 weeks	35 (57)	46	31 days- 10 years^*^	22 (48)
Nelson et al. (9)	35	35	1 month	32 (91)	35	48.4 months	19 (54)
St Peter et al. (40)	35				35	NR	35 (100)
Constantinou et al. (8)	100	100	2 weeks	87 (87)	100	6 months- 5 years^*^	77 (77)
Kaye et al. (26)	75				75	4 months	55 (77)
Hofeldt et al. (41)	30				29	NR	28 (97)
Brownie et al. (29)	41				41	8.4 months	28 (68)
Lyons et al. (30)	37	37	<1 month	23 (62)	30	1 month- 10 years^*^	13 (43)
Johnson et al. (27)	50				50	26.5 weeks	43 (86)
Lacher et al. (5)	82				52	8 months- 5.3 years^*^	23 (44)
Knott et al. (32)	105	105	26 days	70 (76)	56	24.4 months	34 (61)
Srinath et al. (7)	207	183	1 month	129 (71)	190	NR	110 (58)
Mahida et al. (28)	153	153	<2 months	86 (56)	41	25-45 months^*^	18 (44)
Jones et al. (42)	363				NR	NR	NR
Lai et al. (18)	215				181	29.3 weeks	110 (61)
Krishna et al. (17)	236				236	NR	162 (69)
Cairo et al. (14)	49				23	15.4 months	13 (57)
Cairo et al. (16)	678	530	21 days	273 (52)	NR	NR	NR

* Range.

*NR, not reported*.

While the response rate may indicate efficacy for cholecystectomy in children with biliary dyskinesia, there are numerous issues in interpreting the current literature and many important questions which remain unanswered. All published studies are retrospective, non-randomized, and lack control groups and are therefore of low quality. Outcomes are generally not determined in any systematic fashion or utilizing any validated outcome measure and it is unclear if outcomes are self-reported. There is a paucity of long-term outcome data with a significant proportion of patients lost to follow-up when attempts have been made to determine long term response. Even when long term follow-up has been attempted with a standardized questionnaire, there is the risk of recall bias. Another factor inherent in previous studies is selection bias. This is true not only in the decision to publish (and possibly in excluding studies which were not chosen for publication after submission), but in the original clinical decision on whom to operate, as noted in the sections above. There are no standardized pediatric criteria for diagnosing biliary dyskinesia so symptomatic diagnosis and the degree of reliance on tests such as cholescintigraphy are at the discretion of individual clinicians. In bivariate analysis, previous evaluation and referral from a gastroenterologist was shown to have a positive effect on outcome, adding another layer of patient selection (16). In this study, psychologic co-morbidity was shown to have a negative effect on patient outcomes (16). This is similar to what has been reported with children and adolescents with functional dyspepsia and irritable bowel syndrome (43). Psychologic co-morbidity has not been controlled for in previous studies, but certainly may influence decision-making on whom to operate.

It is also not clear if cholecystectomy is superior to no operation or to non-specific symptomatic medical management as there is a paucity of literature in this area. In one of the few available studies, Nelson and colleagues compared outcomes at 2 years in children who underwent cholecystectomy (*N* = 35) as compared to those who did not have cholecystectomy (*N* = 20) (9). In the operative group, 54.3% of patients had complete resolution of pain and 20% had partial resolution. In the non-operative group, 55% of patients had complete resolution of pain and 20% had partial resolution (9). In another study, Kwatra et al. reported on 31 children with an abnormal GBEF (44). Twenty-two had surgery with improvement in 72.7%. Improvement was noted in all who did not have surgery (44). While this could indicate that operative management is not superior to non-operative management, there were likely patient or clinician factors which influenced the decision to operate and which may have influenced relative outcomes given that neither study was a randomized trial. While the lack of randomization poses one set of problems in terms of evaluating outcomes and identifying which patients might obtain greatest benefit from surgery, the inability to blind patients in particular to their management group makes the influence of the placebo-response specifically on surgical outcomes difficult to assess. The placebo-response is known to be a significant factor in therapeutic trials in other FGIDs. In published functional dyspepsia (another FGID) trials in adults, placebo response rates vary from 6 to 72% and in large trials, seem to be stable around 45% (45). Approximately 41% of children with abdominal pain- related functional gastrointestinal disorders demonstrate improvement on placebos (46). A meta-analysis determined that spontaneous improvement and placebo-effect are significant contributors to the therapeutic effect observed with medication (47). The placebo effect is significantly influenced by the relationship between the patient and the clinician (48). Thus, outcomes in individual studies may be influenced by the quality of the patient-physician relationship and patient belief in the efficacy of the particular management approach, in addition to the experience of the surgeon and their individual ability to identify patients who could benefit from cholecystectomy.

## Histopathology

Chronic cholecystitis is common in gallbladders of youth who have had a cholecystectomy for a diagnosis of biliary dyskinesia. Frequencies ranged from 27 to 100% (median 58%) in the studies included in the current review. This may be analogous to the most common FGIDs associated with abdominal pain, functional dyspepsia and irritable bowel syndrome, as chronic inflammation has been implicated in both conditions (49). It is not clear whether chronic inflammation slows gallbladder emptying, gallbladder stasis promotes chronic inflammation, or they are epiphenomena. Jones and colleagues found that GBEF did not differ between patients with and without chronic cholecystitis in a group of patients with biliary dyskinesia (42). In another study, Kwatra et al. evaluated a retrospective cohort of patients with chronic acalculous cholecystitis (CAC) who had a hepatobiliary scan and subsequent cholecystectomy (44). Patients were considered to have CAC if they had abnormal histology (chronic inflammation), no other diagnosis to explain symptoms, and symptoms did not resolve without cholecystectomy. Cholescintigraphy was 95% sensitive and 73% specific, with a negative predictive value of 97.9%, in identifying CAC (44). Their data would suggest that GBEF may be more associated with CAC. It is also possible that chronic cholecystitis and biliary dyskinesia are two distinct conditions without overlap and that the differentiation can only be made definitively after cholecystectomy. Additionally, or alternatively, biliary dyskinesia might be associated with mucosal mast cells, also analogous to what has been reported with functional dyspepsia and irritable bowel syndrome (50, 51). Compared to autopsy controls, we have previously demonstrated a >9-fold increase in gallbladder mast cell density in children with biliary dyskinesia (52). A second prospective study of a different biliary dyskinesia cohort found mast cell density similar to the first study and moderate mast cell degranulation (53). Eighty-five percent of these patients also had chronic cholecystitis. Sharma et al. further demonstrated increased mast cell density in the gallbladder lamina propria and muscularis mucosae of patients with biliary dyskinesia as compared to controls (54). In contrast, Hudson et al. demonstrated greater mast cell density in patients with minimal inflammation as compared to patients with chronic cholecystitis, which could again suggest that cholecystitis may be a distinct process from biliary dyskinesia (55). More research is needed to understand the pathophysiology of biliary dyskinesia.

## Conclusions and Future Directions

There is a lack of consensus regarding the symptom profile defining biliary dyskinesia. While cholescintigraphy appears to be universally used to diagnose biliary dyskinesia, the current literature casts significant doubt on the utility of the test, particularly in its use to select patients for cholecystectomy. Thus, it remains difficult for physicians to predict which patients will achieve long-term benefits from surgery. There are a number of important issues that need to be resolved before continuing with the increasing rate of cholecystectomy in children diagnosed with biliary dyskinesia. Clarity needs to be developed regarding whether biliary dyskinesia should be conceptualized as a functional disorder as it has been in adults, with consensus developed regarding the symptom criteria and the role of cholescintigraphy in defining this condition. Utilization of detailed and consistent protocols for cholescinitgraphy across centers would be helpful in this process. Subsequently, it is vitally important to assess outcomes in a prospective fashion utilizing standardized and validated patient-reported outcome measures in both the short-term and the long-term. Outcomes should be assessed not only for cholecystectomy but for non-surgical interventions currently utilized in the treatment of other functional disorders given some early suggestion of similar pathways at play. These could include placebo-controlled trials of medications directed toward dysmotility (e.g., cisapride or erythromycin), hyperalgesia (e.g., anti-depressants), or inflammation (e.g., mast cell stabilizers) combined with psychosocial approaches (e.g., cognitive-behavioral therapy) which have been found to be effective in other FGIDs. While blinded randomized clinical trials may not be possible, more careful definition and follow up of patients selected for surgical vs. medical management will be important in developing indicators for preferential treatment with each approach. Only with better quality research can we make well-informed decisions about balancing the risks and benefits of surgical vs. medical management of youth with biliary dyskinesia.

## Author Contributions

DS, CF, JS, and JC participated in the literature search, manuscript writing, and critical review of the manuscript.

### Conflict of Interest

The authors declare that the research was conducted in the absence of any commercial or financial relationships that could be construed as a potential conflict of interest.

## References

[1] Cotton PB Elta GH Carter CR Pasricha PJ Corazziari ES Rome IV. Gallbladder and sphincter of Oddi disorders. Gastroenterology. (2016) 150:1420–9e.2. 10.1053/j.gastro.2016.02.033 27144629

[2] Al-Homaidhi HS Sukerek H Klein M Tolia V. Biliary dyskinesia in children. Pediatr Surg Int. (2002) 18:357–60. 10.1007/s00383-002-0822-3 12415355

[3] Ponsky TA DeSagun R Brody F. Surgical therapy for biliary dyskinesia: a meta-analysis and review of the literature. J Laparoendos Adv Surg Tech A. (2005) 15:439–42. 10.1089/lap.2005.15.439 16185113

[4] Preston JF Diggs BS Dolan JP Gilbert EW Schein M Hunter JG. Biliary dyskinesia: a surgical disease rarely found outside the United States. Am J Surg. (2015) 209:799–803. 10.1016/j.amjsurg.2015.01.003 25771131

[5] Lacher M Yannam GR Muensterer OJ Aprahamian CJ Haricharan RN Perger L. Laparoscopic cholecystectomy for biliary dyskinesia in children: frequency increasing. J Pediatr Surg. (2013) 48:1716–21. 10.1016/j.jpedsurg.2012.08.036 23932611

[6] Vegunta RK Raso M Pollock J Misra S Wallace LJ Torres A Jr. Biliary dyskinesia: the most common indication for cholecystectomy in children. Surgery. (2005) 138:726–31. 10.1016/j.surg.2005.06.052 16269302

[7] Srinath AI Youk AO Bielefeldt K. Biliary dyskinesia and symptomatic gallstone disease in children: two sides of the same coin? Dig Dis Sci. (2014) 59:1307–15. 10.1007/s10620-014-3126-2 24715545 PMC4113830

[8] Constantinou C Sucandy I Ramenofsky M. Laparoscopic cholecystectomy for biliary dyskinesia in children: report of 100 cases from a single institution. Am Surg. (2008) 74:587–59. 18646475

[9] Scott Nelson R Kolts R Park R Heikenen J. A comparison of cholecystectomy and observation in children with biliary dyskinesia. J Pediatr Surg. (2006) 41:1894–8. 10.1016/j.jpedsurg.2006.06.018 17101366

[10] Siddiqui S Newbrough S Alterman D Anderson A Kennedy A. Efficacy of laparoscopic cholecystectomy in the pediatric population. J Pediatr Surg. (2008) 43:109–13. 10.1016/j.jpedsurg.2007.09.031 18206466

[11] Matta SR Kovacic K Yan K Simpson P Sood MR. Trends of cholecystectomies for presumed biliary dyskinesia in children in the United States. J Pediatr Gastroenterol Nutr. (2018) 66:808–10. 10.1097/MPG.0000000000001777 29036007

[12] Bielefeldt K Saligram S Zickmund SL Dudekula A Olyaee M Yadav D. Cholecystectomy for biliary dyskinesia: how did we get there? Dig Dis Sci. (2014) 59:2850–63. 10.1007/s10620-014-3342-9 25193389

[13] Walker SK Maki AC Cannon RM Foley DS Wilson KM Galganski LA. Etiology and incidence of pediatric gallbladder disease. Surgery. (2013) 154:927–31. 10.1016/j.surg.2013.04.040 24074432

[14] Cairo SB Ventro G Sandoval E Rothstein DH. Long-term results of cholecystectomy for biliary dyskinesia: outcomes and resource utilization. J Surg Res. (2018) 230:40–6. 10.1016/j.jss.2018.04.044 30100038

[15] Santucci NR Hyman PE Harmon CM Schiavo JH Hussain SZ. Biliary dyskinesia in children: a systematic review. J Pediatr Gastroneterol Nutr. (2017) 64:186–93. 10.1097/MPG.0000000000001357 27472474

[16] Cairo SB Aranda A Bartz-Kurycki M Baxter KJ Bonasso P Dassinger M. Variability in perioperative evaluation and resource utilization in pediatric patients with suspected biliary dyskinesia: a multi-institutional retrospective cohort study. J Pediatr Surg. (2019) 54:1118–22. 10.1016/j.jpedsurg.2019.02.049 30885555 PMC6822378

[17] Krishna YT Griffin KL Gates RL. Pediatric biliary dyskinesia: evaluating predictive factors for successful treatment of biliary dyskinesia with laparoscopic cholecystectomy. Am Surg. (2018) 84:1401–5. 30268165

[18] Lai SW Rothenberg SS Kay SM Shipman KE Slater BJ. Outcomes of laparoscopic cholecystectomy for biliary dyskinesia in children. J Laparoendosc Adv Surg Tech A. (2017) 27:845–50. 10.1089/lap.2016.0338 28350202

[19] Milinić N Filipovic B Lukić T Marković O Milisavljević N Gajić M. Ultrasonography analysis of gallbladder motility in patients with functional dyspepsia. Eur J Intern Med. (2014) 25:156–9. 10.1016/j.ejim.2013.08.699 24012325

[20] Sood GK Baijal SS Lahoti D Broor SL. Abnormal gallbladder function in patients with irritable bowel syndrome. Am J Gastroenterol. (1993) 88:1387–90. 8362836

[21] Veras Neto MC Yamada RM da Costa Pinto EA. Gallbladder motility in children with chronic constipation. J Pediatr Gastroenterol Nutr. (2008) 46:414–8. 10.1097/MPG.0b013e31813347c4 18367954

[22] Delgado-Aros S Cremonini F Bredenoord AJ Camilleri M. Systematic review and meta-analysis: does gall-bladder ejection fraction on cholecystokinin cholescintigraphy predict outcome after cholecystectomy in suspected functional biliary pain? Aliment Pharmacol Ther. (2003) 18:167–74. 10.1046/j.1365-2036.2003.01654.x 12869076

[23] DiBaise JK Oleynikov D. Does gallbladder ejection fraction predict outcome after cholecystectomy for suspected chronic acalculous gallbladder dysfunction? A systematic review. Am J Gastroenterol. (2003) 98:2605–11. 10.1111/j.1572-0241.2003.08772.x 14687804

[24] Gudsoorkar VS Oglat A Jain A Raza A Quigley EMM. Systematic review with meta-analysis: cholecystectomy for biliary dyskinesia- what can the gallbladder ejection fraction tell us? Aliment Pharmacol Ther. (2019) 49:654–63. 10.1111/apt.15128 30706496

[25] Pihl KD Jones MW Deppen JG Ferguson TM Hanses SM. Effects of laparoscopic cholecystectomy in normokinetic biliary dyskinesia. Am J Surg. (2018) 215:116–9. 10.1016/j.amjsurg.2017.04.012 28669533

[26] Kaye AJ Jatla M Mattei P Kelly J Nance ML. Use of laparoscopic cholecystectomy for biliary dyskinesia in the child. J Pediatr Surg. (2008) 43:1057–9. 10.1016/j.jpedsurg.2008.02.034 18558182

[27] Johnson JJ Garwe T Katseres N Tuggle DW. Preoperative symptom duration predicts success in relieving abdominal pain caused by biliary dyskinesia in a pediatric population. J Pediatr Surg. (2013) 48:796–800. 10.1016/j.jpedsurg.2012.10.047 23583136

[28] Mahida JB Sulkowski JP Cooper JN King AP Deans KJ King DR. Prediction of symptom improvement in children with biliary dyskinesia. J Surg Res. (2015) 198:393–9. 10.1016/j.jss.2015.03.056 25891671

[29] Brownie E Cusick RA Perry DA Allbery S Azarow KS. Pathologic changes in biliary dyskinesia. J Pediatr Surg. (2011) 46:879–82. 10.1016/j.jpedsurg.2011.02.021 21616245

[30] Lyons H Hagglund KH Smadi Y. Outcomes after laparoscopic cholecystectomy in children with biliary dyskinesia. Surg Laparosc Endosc Percutan Tech. (2011) 21:175–8. 10.1097/SLE.0b013e31821db7b2 21654301

[31] Carney DE Kokoska ER Grosfeld JL Engum SA Rouse TM West KM. Predictors of successful outcome after cholecystectomy for biliary dyskinesia. J Pediatr Surg. (2004) 39:813–6. 10.1016/j.jpedsurg.2004.02.017 15185202

[32] Knott EM Fike FB Gasior AC Cusick R Brownie E St Peter SD. Multi-institutional analysis of long-term symptom resolution after cholecystectomy for biliary dyskinesia in children. Pediatr Surg Int. (2013) 29:1243–7. 10.1007/s00383-013-3343-3 23846453

[33] Chua ASB Keeling PWN. Cholecystokinin hyperresponsiveness in functional dyspepsia. World J Gastroenterol. (2006) 12:2688–93. 10.3748/wjg.v12.i17.2688 16718754 PMC4130976

[34] Benini F Mora A Turini D Bertolazzi S Lanzarotto F Ricci C. Slow gallbladder emptying reverts to normal but small intestinal transit of a physiological meal remains slow in celiac patients during gluten-free diet. Neurogastroenterol Motil. (2012) 24:100–7. 10.1111/j.1365-2982.2011.01822.x 22097920

[35] Cahan M Balduf L Colton K Palacioz B McCartney W Farrell TM. Proton pump inhibitors reduce gallbladder function. Surg Endosc. (2006) 20:1364–7. 10.1007/s00464-005-0247-x 16858534

[36] Dhiman RK Arke L Bhansali A Gupta S Chawla YK. Cisapride improves gallbladder emptying in patients with type 2 diabetes mellitus. J Gastroenterol Hepatol. (2001) 16:1044–50. 10.1046/j.1440-1746.2001.02586.x 11595071

[37] Catnach SM Ballinger AB Stevens M Fairclough PD Trembath RC Drury PL. Erythromycin induces supranormal gall bladder contraction in diabetic autonomic neuropathy. Gut. (1993) 34:1123–7. 10.1136/gut.34.8.1123 8174966 PMC1374367

[38] Pons V Sopena R Hoyos M Garrigues V Cano C Nos P. Quantitative cholescintigraphy: selection of random dose for CCK-33 and reproducibility of abnormal results. J Nucl Med. (2003) 44:446–50. 12621013

[39] Rose JB Fields RC Strasberg SM. Poor reproducibility of gallbladder ejection fraction by biliary scintigraphy for diagnosis of biliary dyskinesia. J Am Coll Surg. (2018) 226:155–9. 10.1016/j.jamcollsurg.2017.10.025 29157795

[40] St Peter SD Keckler SJ Nair A Andrews WS Sharp RJ Snyder CL. Laparoscopic cholecystectomy in the pediatric population. J. Laparoendosc Adv Surg Tech A. (2008) 18:127–30. 10.1089/lap.2007.0150 18266591

[41] Hofeldt M Richmond B Huffman K Nestor J Maxwell D. Laparoscopic cholecystectomy for treatment of biliary dyskinesia is safe and effective in the pediatric population. Am Surg. (2008) 74:1069–72. 19062663

[42] Jones PM Rosenman MB Pfefferkorn MD Rescorla FJ Bennett WE Jr. Gallbladder ejection fraction is unrelated to gallbladder pathology in children and adolescents. J Pediatr Gastroenterol Nutr. (2016) 63:71–5. 10.1097/MPG.0000000000001065 26670710

[43] Deacy AD Friesen CA Staggs VS Schurman JV. Evaluation of clinical outcomes in an interdisciplinary abdominal pain clinic: a retrospective, exploratory review. World J Gastroenterol. (2019) 25:3079–90. 10.3748/wjg.v25.i24.3079 31293343 PMC6603811

[44] Kwatra NS Nurko S Stamoulis C Falone AE Grant FD Treves ST. Chronic acalculous cholecystitis in children with biliary symptoms: Usefulness of hepatocholescintigraphy. J Pediatr Gastroentrol Nutr. (2019) 68:68–73. 10.1097/MPG.0000000000002151 30256266

[45] Enck P Klosterhalfen S. The placebo response in functional bowel disorders: perspectives and putative mechanisms. Neurogastroenterol Motil. (2005) 17:325–31. 10.1111/j.1365-2982.2005.00676.x 15916619

[46] Hoekman DR Zeevenhooven J van Etten-Jamaludin FS Douwes Dekker I Benninga MA Tabbers MM. The placebo response in pediatric abdominal pain-related functional gastrointestinal disorders: a systematic review and meta-analysis. J Pediatr. (2017) 182:155–63.e7. 10.1016/j.jpeds.2016.12.022 28081889

[47] Krogsbøll LT Hróbjartsson A Gøtzsche PC Spontaneous improvement in randomized clinical trials: meta-analysis of three-armed trials comparing no treatment, placebo and active intervention. BMC Med Res Methodol. (2009) 9:1 10.1186/1471-2288-9-1 19123933 PMC2628943

[48] Benninga MA Mayer EA. The power of placebo in pediatric functional gastrointestinal disease. Gastroenterology. (2009) 137:1207–10. 10.1053/j.gastro.2009.08.023 19717127

[49] B Biomed GB Carroll G Mathe A Horvat J Foster P Walker MM Evidence for local and systemic immune activation in functional dyspepsia and the irritable bowel syndrome: a systematic review. Am J Gastroenterol. (2019) 114:429–36. 10.1038/s41395-018-0377-0 30839392

[50] Du L Chen B Kim JJ Chen X Dai N. Micro-inflammation in functional dyspepsia: a systematic review and meta-analysis. Neurogastroenterol Motil. (2018) 30:e13304. 10.1111/nmo.13304 29392796

[51] Friesen CA Schurman JV Colombo JM Abdel-Rahman SM. Eosinophils and mast cells as therapeutic targets in pediatric functional dyspepsia. World J Gastrointest Pharmacol Ther. (2013) 4:86–96. 10.4292/wjgpt.v4.i4.86 24199024 PMC3817289

[52] Rau B Friesen CA Daniel JF Qadeer A You-Li D Roberts CC. Gallbladder wall inflammatory cells in pediatric patients with biliary dyskinesia and cholelithiasis: a pilot study. J Pediatr Surg. (2006) 41:1545–8. 10.1016/j.jpedsurg.2006.05.015 16952589

[53] Friesen CA Neilan N Daniel JF Radford K Schurman JV Li DY. Mast cell activation and clinical outcome in pediatric cholelithiasis and biliary dyskinesia. BMC Research Notes. (2011) 4:322. 10.1186/1756-0500-4-322 21896203 PMC3224507

[54] Sharma SG Saad AG Biliary dyskinesia in children: a mast cell-related disorder? Lab Invest. (2011) 91:403A.

[55] Hudson I Hopwood D. Macrophages and mast cells in chronic cholecystitis and normal gall bladders. J Clin Pathol. (1986) 39:1082–7. 10.1136/jcp.39.10.1082 2431004 PMC500226

